# Supplementary material for: Focus on the spectra that matter by clustering of quantification data in shotgun proteomics
Source: Nat Commun. 2020 Jun 26;11:3234. doi: 10.1038/s41467-020-17037-3 (PMC7319958; doi:10.1038/s41467-020-17037-3)
Supplement: Supplementary file 1 — Supplementary Information [file 41467_2020_17037_MOESM1_ESM.pdf]

Supplement to: "Focus on the spectra that matter by clustering of quantification data in shotgun proteomics"

Matthew The[1] and Lukas Käll[1]

[1]Science for Life Laboratory, KTH – Royal Institute of Technology, Box 1031, 17121 Solna, Sweden, Correspondence and requests for materials should be addressed to L.K. , (email: lukas.kall@scilifelab.se)

May 18, 2020

Supplementary Note 1

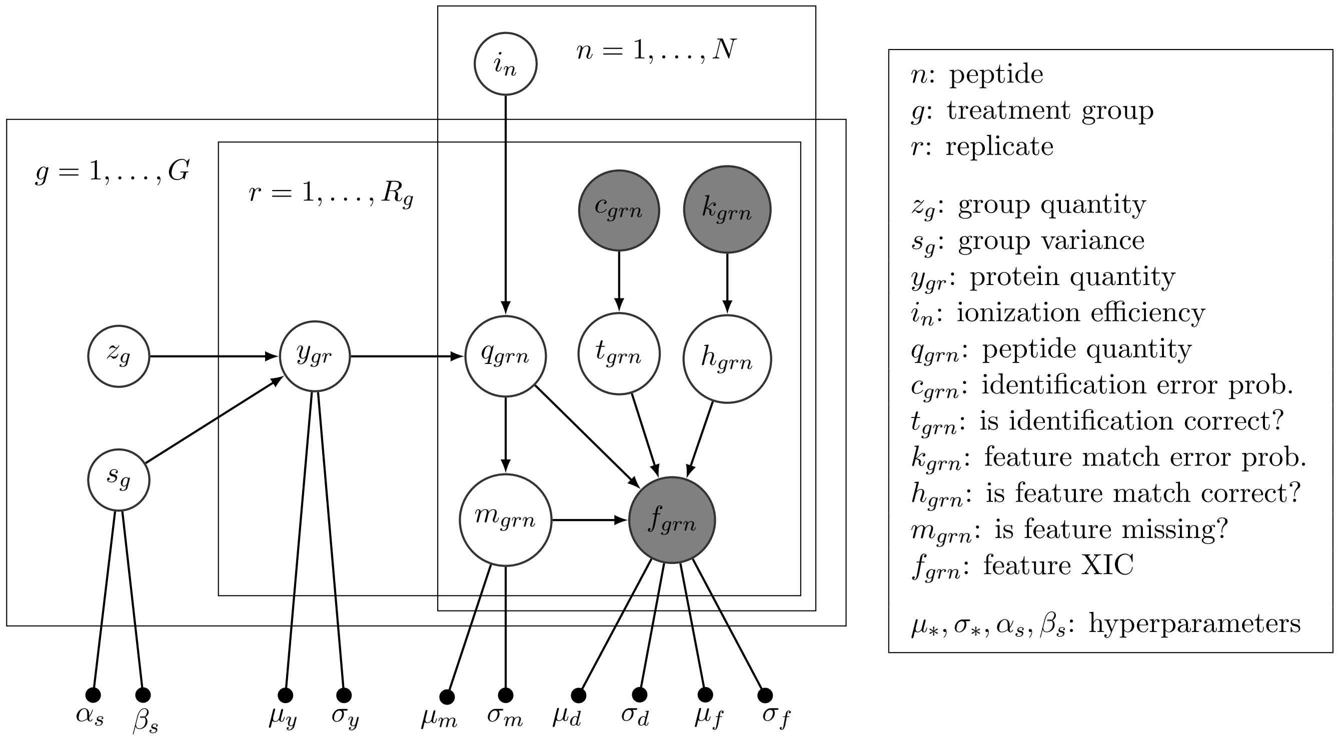

Supplementary Figure 1: **Updated probabilistic graphical model for Triqler with k_{grn} and h_{grn} nodes for feature-feature match PEPs.** The protein has N peptides, G treatment groups and R_g replicates per treatment group. Gray nodes denote observed variables, whereas white nodes denote latent variables. A concrete example is presented in section .

For the new indicator variable h_{grn} we define:

$$h_{grn} = \begin{cases} 0, & \text{if feature match is correct} \\ 1, & \text{if feature match is incorrect} \end{cases}$$

The term $p(f_{grn} \mid q_{grn}, t_{grn}, m_{grn})$ is now replaced by $p(f_{grn} \mid q_{grn}, t_{grn}, m_{grn}, h_{grn})$, which is defined

by:

$$
\begin{aligned}
p(f_{grn} = \text{NaN} \mid m_{grn} = 0, t_{grn} = 0) &= 0 \\
p(f_{grn} = \text{NaN} \mid q_{grn} = a, m_{grn} = 1, t_{grn} = 0, h_{grn} = 0) &= 1 \\
p(f_{grn} = \text{NaN} \mid h_{grn} = 1) = p(f_{grn} = \text{NaN} \mid t_{grn} = 1, h_{grn} = 0) &= \text{sigm}(w; \mu_m, \sigma_m) \\
p(f_{grn} = x \mid m_{grn} = 1, t_{grn} = 0) &= 0 \\
p(f_{grn} = x \mid q_{grn} = a, m_{grn} = 0, t_{grn} = 0, h_{grn} = 0) &= \text{hypsec}(a - x; \mu_d, \sigma_d) \\
p(f_{grn} = x \mid h_{grn} = 1) = p(f_{grn} = x \mid t_{grn} = 1, h_{grn} = 0) &= (1 - \text{sigm}(w; \mu_m, \sigma_m)) \cdot \text{hypsec}(w - x; \mu_d, \sigma_d)
\end{aligned}
$$

with $w = \left(\prod_W f_{g'r'n}\right)^{1/|W|}$ and $W = \{(g', r') \mid (g', r') \neq (g, r), f_{g'r'n} \neq \text{NaN}\}$, the geometric mean of all non-missing other features from the same feature group.

For the term $p(h_{grn} \mid k_{grn})$ we simply have

$$
\begin{aligned}
p(h_{grn} = 1 \mid k_{grn}) &= k_{grn} \\
p(h_{grn} = 0 \mid k_{grn}) &= 1 - k_{grn}
\end{aligned}
$$

Supplementary Note 2

As an **example of Triqler summarization**, let us consider one of the UPS proteins, P08263ups|GSTA1_HUMAN_UPS, from the UPS-Yeast mix dataset. This protein has 4 peptide identifications and, ideally, we want to show that this protein is present in different concentrations in each of the 3 groups, where the UPS proteins were spiked in at 5, 10 and 25 fmol.

For each combination of sample and peptide, we have 3 pieces of information: the feature intensity (XIC, f_{grn}), the posterior probability of the MS2 cluster identification to be incorrect (P_{id}, c_{grn}) and the posterior probability of the MS1 feature to be incorrectly matched to one of the MS1 features with an MS2 cluster identification (P_{match}, k_{grn}):

We can plot the posterior distributions on several levels for this particular protein using some extra functionality of the Triqler python package:

```
python -m triqler.distribution.plot_posteriors --protein_id P08263ups
    --fold_change_eval 0.8 --decoy_pattern decoy_ --max_plot_fold_change 4.0
    --spike_in_concentrations 25,10,5
    UPS_Yeast_mix.quant_rows.tide_concat_peptides.tsv
```

On protein level (Supplementary Figure 2), we can see the influence of the missing values in C-25fmol-R3 (first pane, red series) and D-10fmol-R1 (second pane, blue series). These missing values shift some of the probability to lower relative protein quant levels, but due to the strong evidence from the other peptides, this shift is only modest. Also, note that these posteriors are not necessarily normal distributions.

On experimental group level (Supplementary Figure 3), we can still see traces of the contributions of the individual proteins, especially in the different widths of the group distributions. However, due to the averaging over the three replicates, we end up with distributions that are very close to normal distributions.

On fold change level (Supplementary Figure 4, note that the abundance scale changed from $\log_{10}$ to $\log_2$ compared to the treatment group posterior plots), the width of the distributions for 25 vs 10 is noticably smaller than for the other two comparisons, which can be attributed to the larger uncertainty in abundance for the 5 fmol protein and group distributions.

Some other notable features of the graphical model:

group	25 fmol ($g = 1$)								
P08263ups|GSTA1_HUMAN_UPS	C-25fmol-R1 ($r = 1$)			C-25fmol-R2 ($r = 2$)			C-25fmol-R3 ($r = 3$)		
	XIC f_{11n}	P_{id} c_{11n}	P_{match} k_{11n}	XIC f_{12n}	P_{id} c_{12n}	P_m k_{12n}	XIC f_{13n}	P_{id} c_{13n}	P_m k_{13n}
ISNLPTVK ($n = 1$)	61.9	0.004	0.027	73.0	0.003	0	NaN	0.004	0.066
YFPAFEK ($n = 2$)	103.5	0.02	0	92.2	0.02	0	95.1	0.02	0
KFLQPGSPRKPPMDEK ($n = 3$)	0.12	0.93	0.04	0.20	0.93	0.02	0.24	0.93	0.07
FLQPGSPR ($n = 4$)	66.3	0.028	0	55.4	0.028	0	84.3	0.028	0

group	10 fmol ($g = 2$)								
P08263ups|GSTA1_HUMAN_UPS	D-10fmol-R1 ($r = 1$)			D-10fmol-R2 ($r = 2$)			D-10fmol-R3 ($r = 3$)		
	XIC f_{21n}	P_{id} c_{21n}	P_{match} k_{21n}	XIC f_{22n}	P_{id} c_{22n}	P_m k_{22n}	XIC f_{23n}	P_{id} c_{23n}	P_m k_{23n}
ISNLPTVK ($n = 1$)	7.56	0.004	0.049	20.6	0.004	0	18.0	0.004	0.054
YFPAFEK ($n = 2$)	NaN	0.02	0.017	22.0	0.02	0.012	26.6	0.02	0
KFLQPGSPRKPPMDEK ($n = 3$)	0.23	0.93	0.020	0.13	0.93	0.033	0.11	0.93	0.015
FLQPGSPR ($n = 4$)	16.7	0.028	0.15	15.0	0.028	0.12	19.53	0.028	0.20

group	5 fmol ($g = 3$)								
P08263ups|GSTA1_HUMAN_UPS	E-5fmol-R1 ($r = 1$)			E-5fmol-R2 ($r = 2$)			E-5fmol-R3 ($r = 3$)		
	XIC f_{31n}	P_{id} c_{31n}	P_{match} k_{31n}	XIC f_{32n}	P_{id} c_{32n}	P_m k_{32n}	XIC f_{33n}	P_{id} c_{33n}	P_m k_{33n}
ISNLPTVK ($n = 1$)	6.96	0.004	0.066	7.27	0.004	0	2.38	0.004	0.009
YFPAFEK ($n = 2$)	4.81	0.02	0.017	1.83	0.02	0.010	8.68	0.02	0.007
KFLQPGSPRKPPMDEK ($n = 3$)	0.14	0.93	0	0.39	0.93	0	0.13	0.93	0.009
FLQPGSPR ($n = 4$)	10.9	0.028	0.09	5.36	0.028	0.077	10.9	0.028	0.055

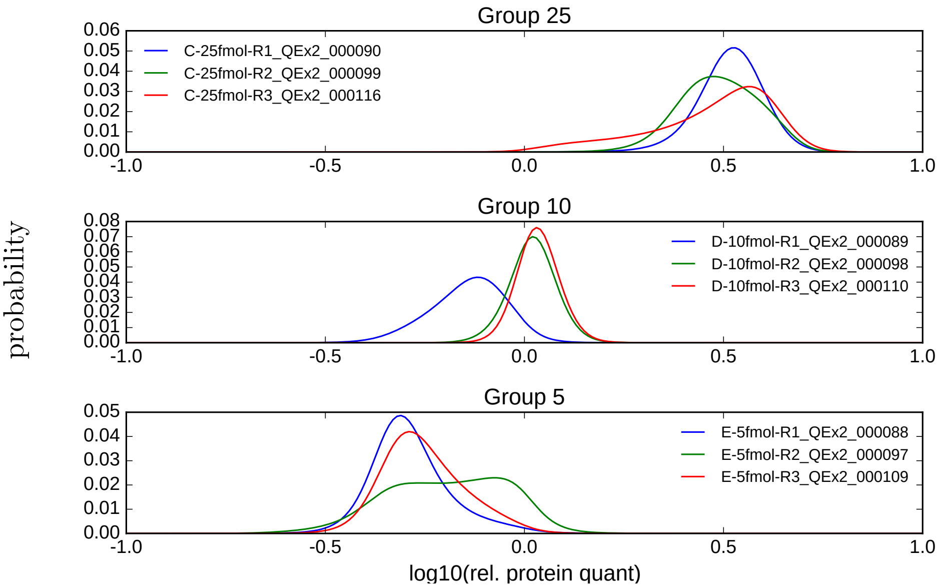

Supplementary Figure 2: **Posterior distributions for protein abundances as calculated by Triqler.** We plot the probabilities as a function of the $\log_{10}$ relative protein abundance for each of the 9 samples, grouped by spike-in concentration. We can see some clear contributions of the individual peptides, such as missing values (Group 10, blue series) and disagreement between different peptides (Group 5, green series).

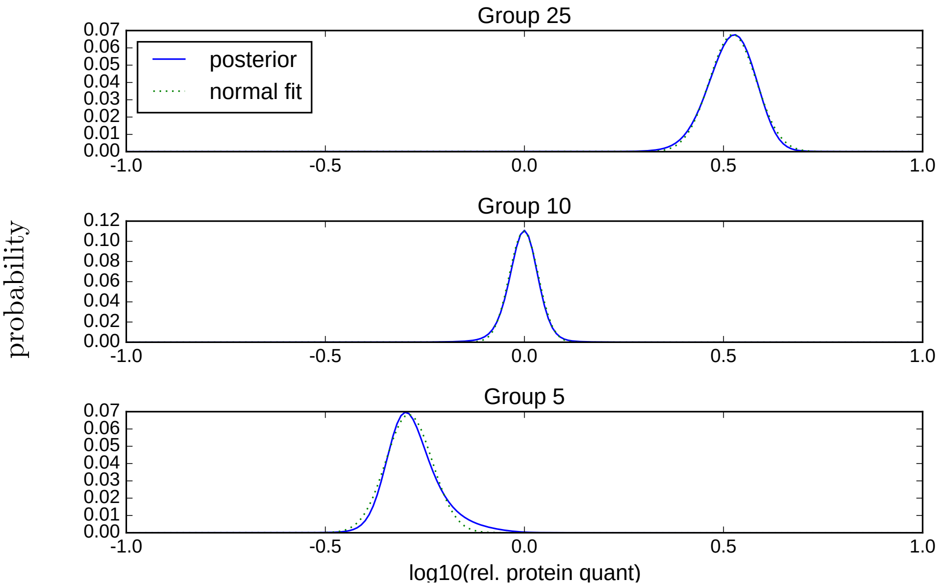

Supplementary Figure 3: **Posterior distributions for experimental group mean abundances as calculated by Triqler.** We plot the probabilities as a function of the $\log_{10}$ experimental group mean abundance for each of the 3 spike-in concentrations. Group 10 clearly has a narrower distribution, owing to the better agreement between peptides and replicates. For illustration puposes, we have included least square fittings of normal distributions to the calulated posterior distributions.

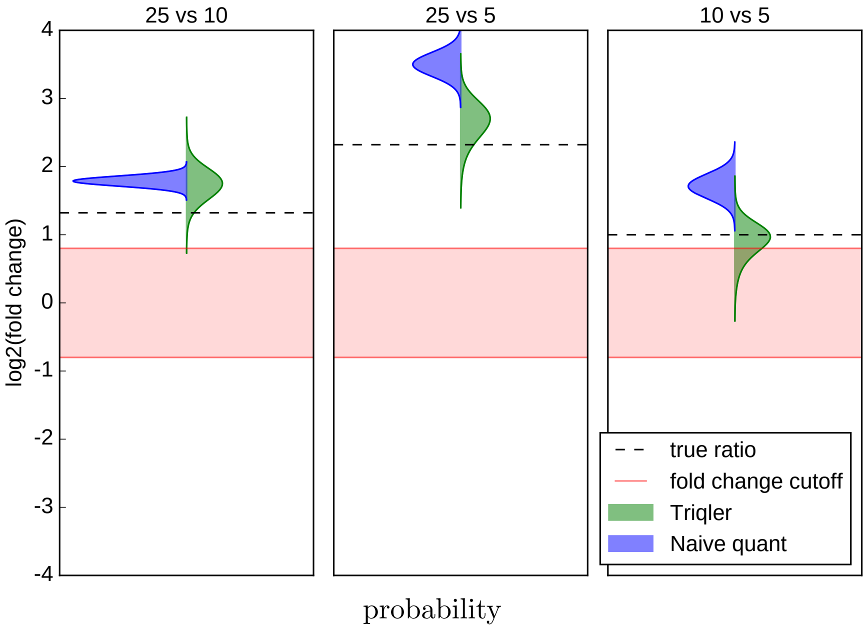

Supplementary Figure 4: **Posterior distributions for fold changes between experimental groups as calculated by Triqler (green) and a naive quantification method (blue).** We plot the probabilities as a function of the $\log_2$ fold change between experimental groups. The naive quantification method summed up the 3 most intense peptides and imputed missing values by the row mean. We plot the naive quantification method's results as normal distributions using the mean and standard error of the mean. Triqler manages to produce posterior distributions that are closer to the true ratios.

- For the samples that have an MS2 spectrum in the MS2 spectrum cluster that identified the peptide in question, the feature match error probability P_m is 0, e.g. k_{121}. This neglects the possibility that the MS2 spectrum was erroneously assigned to the MS1 feature group or that the MS2 spectrum was erroneously assigned to the MS2 spectrum cluster. We assume these probabilities to be small, but incorporating these in an extension of the model might be addressed in future work.
- Conversely, a non-zero P_m value, e.g. k_{111}, means that the sample in question did not have an MS2 spectrum that could be associated to the peptide but did have an MS1 feature that could be matched to one of the MS1 features in another sample in the minimum spanning tree alignment.
- Usually, only one MS2 spectrum cluster will yield a confident peptide identification for an MS1 feature group. In this example, we can see this for YFPAFEK, KFLQPGSPRKPPMDEK and FLQPGSPR, where the identification probability is the same across all samples. In the case of ISNLPTVK, there were 2 MS2 spectrum clusters with the same peptide identification, one with $P_{id} = 0.003$ and one with $P_{id} = 0.004$. This happens if the MS2 spectra are not sufficiently similar for MaRaCluster to cluster them together, for example due to chimericity.
- The MS2 spectrum cluster for KFLQPGSPRKPPMDEK does not have a reliable peptide identification ($P_{id} = 0.93$), and therefore its contribution to the protein quantification will be penalized.
- Both f_{131} and f_{212} are missing values. Combined with the high XIC of f_{111} and f_{121}, the model will, for example, infer that f_{131} is missing completely at random.
- The feature match probability k_{234} for the MS1 feature with XIC 19.53 is rather high. This will penalize the contribution of f_{234}, but as it actually agrees with the other confidently identified peptides its contribution is still important to .

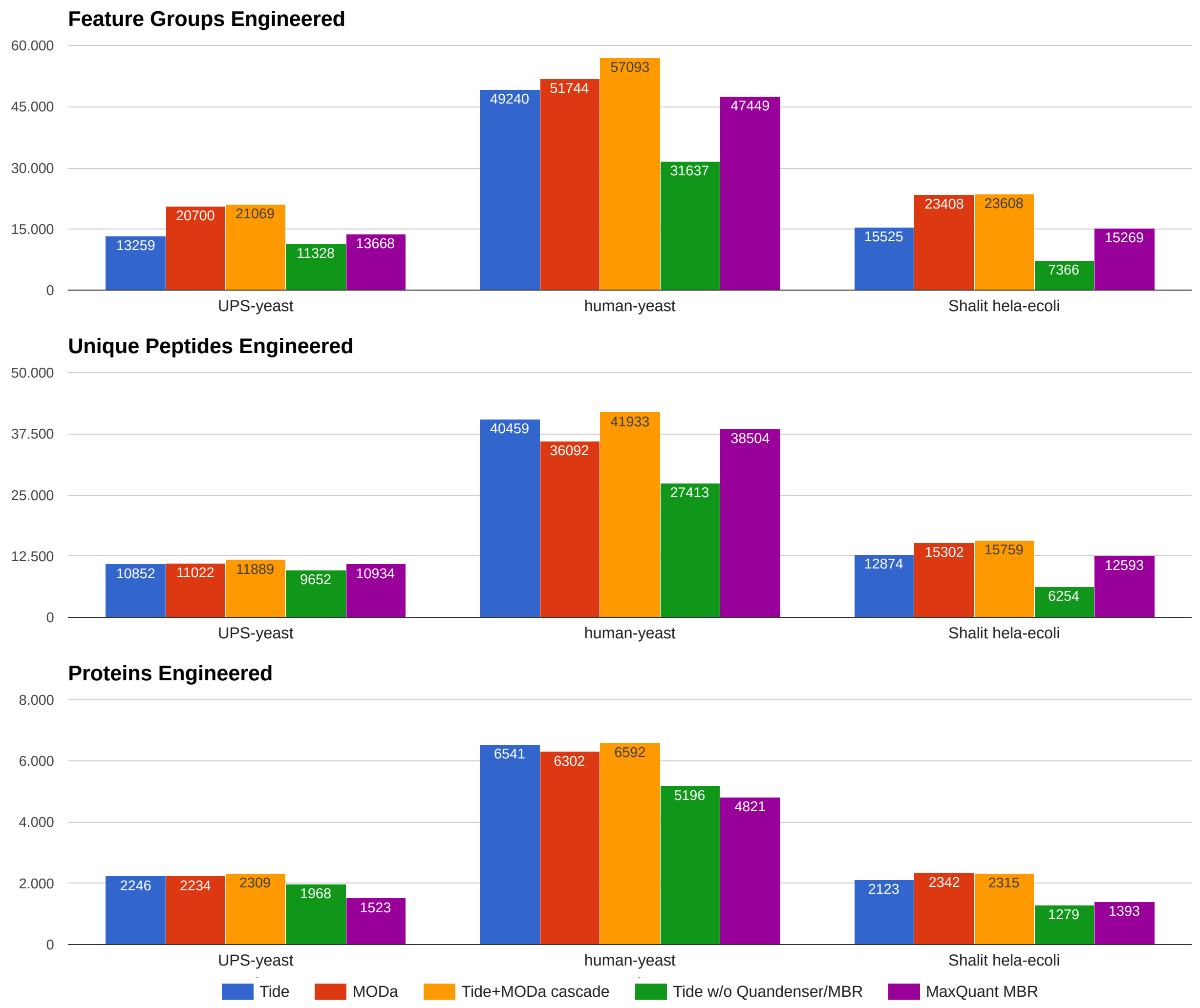

Supplementary Figure 5: **Using a cascaded search with an open modification search as second search increases the number of identifications on all levels on three engineered datasets.** The analyzed methods were Quandenser+Triqler when using the search engine Tide (**blue**), MODa (**red**), and a cascade search of first Tide and subsequently MODa (**yellow**); Tide and Triqler without MaRaCluster and the matches-between-runs (MBR) feature (**green**) and MaxQuant with MBR followed by statistical analysis with Perseus (**orange**). A comparison of the number of feature groups with a peptide identification, unique peptides and proteins at 1% identification FDR shows the superior performance of using a cascaded search which includes an open modification search through MODa. It should be noted that MaxQuant requires at least two unique peptides for protein identification and therefore the difference in the number of identifications on protein level cannot directly be compared.

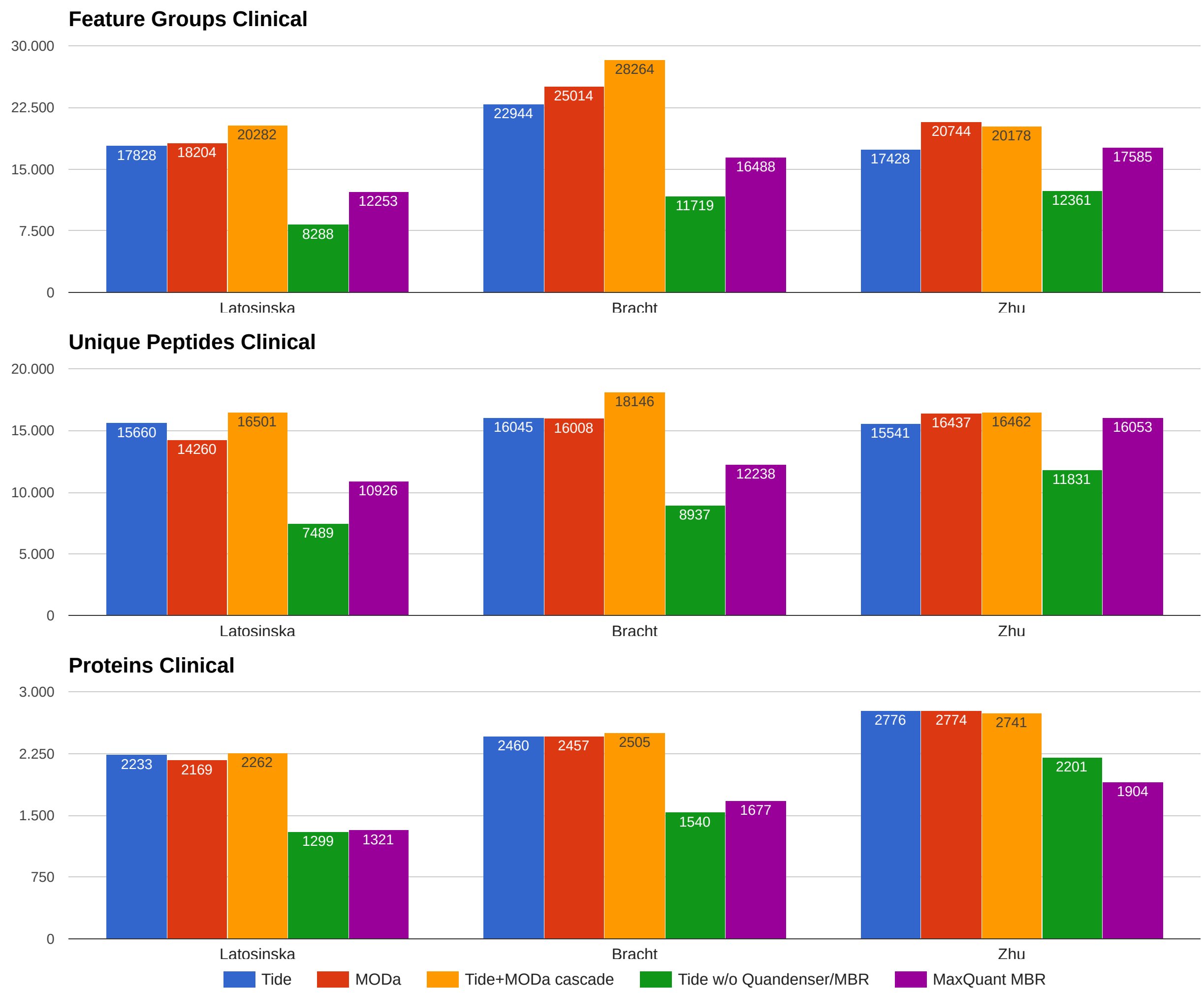

Supplementary Figure 6: **Using a cascaded search with an open modification search as second search increases the number of identifications on feature group and peptide levels for three clinical datasets.** The analyzed methods were Quandenser+Triqler when using the search engine Tide (**blue**), MODa (**red**), and a cascade search of first Tide and subsequently MODa (**yellow**); Tide and Triqler without MaRaCluster and the matches-between-runs (MBR) feature (**green**) and MaxQuant with MBR followed by statistical analysis with Perseus (**orange**). A comparison of the number of feature groups with a peptide identification, unique peptides and proteins at 1% identification FDR shows the superior performance of using a cascaded search which includes an open modification search through MODa. It should be noted that MaxQuant requires at least two unique peptides for protein identification and therefore the difference in the number of identifications on protein level cannot directly be compared.

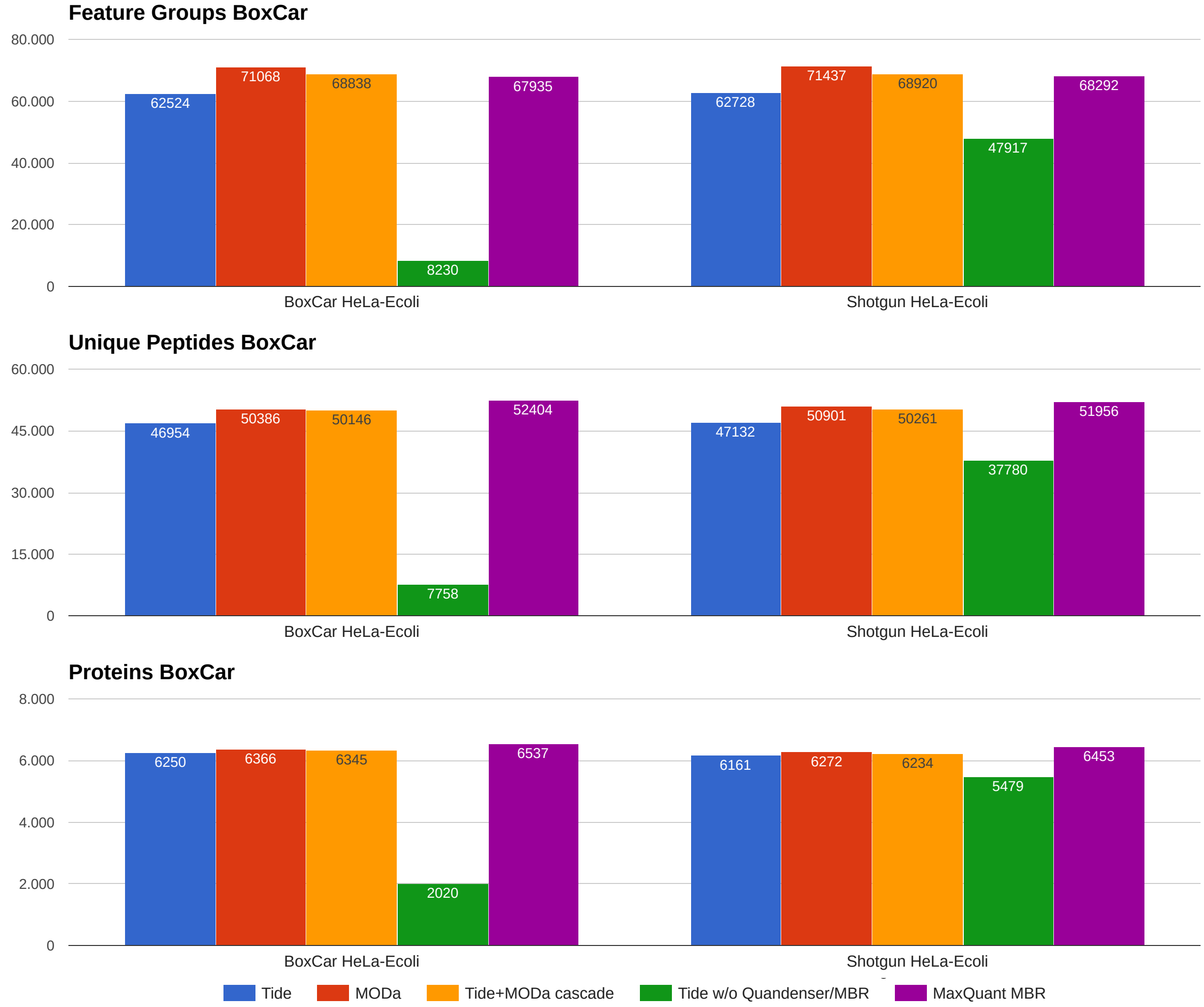

Supplementary Figure 7: **Using a cascaded search with an open modification search as second search increases the number of identifications on all levels for both the Shotgun and BoxCar runs from the BoxCar manuscript.** The analyzed methods were Quandenser+Triqler when using the search engine Tide (**blue**), MODa (**red**), and a cascade search of first Tide and subsequently MODa (**yellow**); Tide and Triqler without MaRaCluster and the matches-between-runs (MBR) feature (**green**) and MaxQuant with MBR followed by statistical analysis with Perseus (**orange**). A comparison of the number of feature groups with a peptide identification, unique peptides and proteins at 1% identification FDR shows the superior performance of using a cascaded search which includes an open modification search through MODa. It should be noted that we used the MaxQuant output files from the original BoxCar manuscript, in which the database contained all proteoforms in Swiss-Prot and TrEMBL, whereas we only searched against the proteins in Swiss-Prot. Therefore, the difference in the number of identifications between MaxQuant and Quandenser+Triqler cannot directly be compared. Also, since the BoxCar approach only samples very few MS2 spectra, peptide identifications need to be inferred using MBR with respect to the Shotgun runs. This explains the poor behavior of Tide without Quandenser/MBR on the BoxCar runs and its decent behavior on the Shotgun runs.

UPS-yeast

UPS1 spike-in concentration [fmol]	25 vs 10		25 vs 5		10 vs 5	
Method	tp	fp	tp	fp	tp	fp
**Quandenser+Tide+Triqler**	**43**	**1**	**45**	**1**	**35**	**0**
MaxQuant MBR+Perseus						
- $S_0 = 0.0$	37	42	35	124	24	7
- $S_0 = 0.3$	39	9	42	15	36	1
- $S_0 = 0.7$	34	9	40	6	24	1
- $S_0 = 1.0$	28	2	41	5	1	0
MaxQuant MBR+EBRCT	43	1	40	9	0	0

Shalit hela-ecoli

E. coli spike-in concentration [ng]	3 vs 7.5		3 vs 10		3 vs 15		7.5 vs 10		7.5 vs 15		10 vs 15	
Method	tp	fp	tp	fp	tp	fp	tp	fp	tp	fp	tp	fp
**Quandenser+Tide+Triqler**	**190**	**7**	**198**	**4**	**229**	**0**	**0**	**0**	**194**	**3**	**138**	**0**
MaxQuant MBR+Perseus												
- $S_0 = 0.0$	75	5	96	38	129	246	0	1	100	27	41	2
- $S_0 = 0.3$	126	7	134	6	137	9	0	0	128	4	125	7
- $S_0 = 0.7$	117	7	134	4	137	7	0	0	125	6	4	0
- $S_0 = 1.0$	114	7	133	4	137	6	0	0	69	5	2	0
MaxQuant MBR+EBRCT	119	12	124	13	130	20	1	1	91	2	28	1

human-yeast

yeast spike-in protein concentration [%]	10 vs 5		10 vs 3.3		5 vs 3.3	
Method	tp	fp	tp	fp	tp	fp
**Quandenser+Triqler**						
**- Tide**	**366**	**24**	**419**	**24**	**198**	**0**
- MODa	223	11	296	15	77	0
- Tide + MODa cascade	334	13	382	20	154	0
Tide+Triqler (without Quandenser/MBR)	188	3	211	11	99	0
MaxQuant MBR+Perseus						
- $S_0 = 0.0$	226	36	266	32	133	22
- $S_0 = 0.3$	322	34	361	27	72	5
- $S_0 = 0.7$	289	27	358	25	1	0
- $S_0 = 1.0$	188	18	356	21	1	0
- original study	296	?	287	?	107	?
MaxQuant MBR+EBRCT	372	41	388	101	28	9

BoxCar hela-ecoli

E. coli spike-in ratio [1 : x]	BoxCar 2 vs 12				Shotgun 2 vs 12			
	1-sided test		2-sided test		1-sided test		2-sided test	
**Method**	tp	fp	tp	fp	tp	fp	tp	fp
**Quandenser+Triqler**								
**- Tide**	**977**	**7**	**977**	**32**	**964**	**10**	**964**	**218**
- MODa	946	18	946	36	950	17	950	216
- Tide + MODa cascade	961	6	961	27	959	6	959	207
Tide+Triqler (without Quandenser/MBR)*	140	4	140	11	743	11	743	59
MaxQuant MBR+Perseus**								
- Student t-test $S_0 = 0.0$	965	16	971	3486	716	21	723	2228
- Welch t-test $S_0 = 0.3$	972	19	975	2452	723	19	723	716

* BoxCar samples very few MS2 spectra; peptide identities are typically inferred with MBR to Shotgun runs, explaining the low sensitivity of the BoxCar runs without any form of MBR

** We used the MaxQuant output files from the original study where the data was searched against Swiss-Prot+TrEMBL, whereas we searched only against Swiss-Prot

Supplementary Table 1: The table lists the number of true and false positive significantly differentially abundant proteins at a 5% reported FDR threshold for (1) Quandenser+Triqler, (2) for different values of S_0 for Welch's t-test in Perseus and (3) for the EBRCT method. Some results for Quandenser+Triqler and MaxQuant+EBRCT are left out here as they are listed in the main manuscript.

Supplementary Note 3

Posterior distributions for fold changes. We plotted the posterior distributions of fold changes as calculated by Triqler for the UPS-Yeast set (Supplementary Figure 8) and the Human+Yeast and HeLa+Ecoli sets (Supplementary Figure 9). We compared these to the results of MaxQuant+Perseus, where we instead of the posterior distribution plot the confidence interval using the mean and standard error of the mean.

Triqler achieves posterior estimates that approximate the true ratios quite well for a large proportion of the spiked-in proteins. Note that proteins with low FDR (towards the top of each panel) show fold changes very close to the expected value, but proteins with higher FDR (towards the bottom of each panel) show fold changes close to 0. The reason for the shift is that the proteins with higher FDR either have 1) peptides with high FDR, which have a harder time to overrule the prior, or 2) incorrect peptides that actually belong to the background. This is exactly the way that you would like a method to behave. You do not want to report inaccurately identified proteins as differentially abundant, i.e. having a fold change far from 0.

MaxQuant+Perseus achieves good quantitative accuracy and narrow confidence regions for highly confident proteins. However, we also see that many proteins for MaxQuant+Perseus have either large variance estimates, which often prevents t-tests from calling significance, or are not quantified because they had fewer than 2 peptides below 1% FDR.

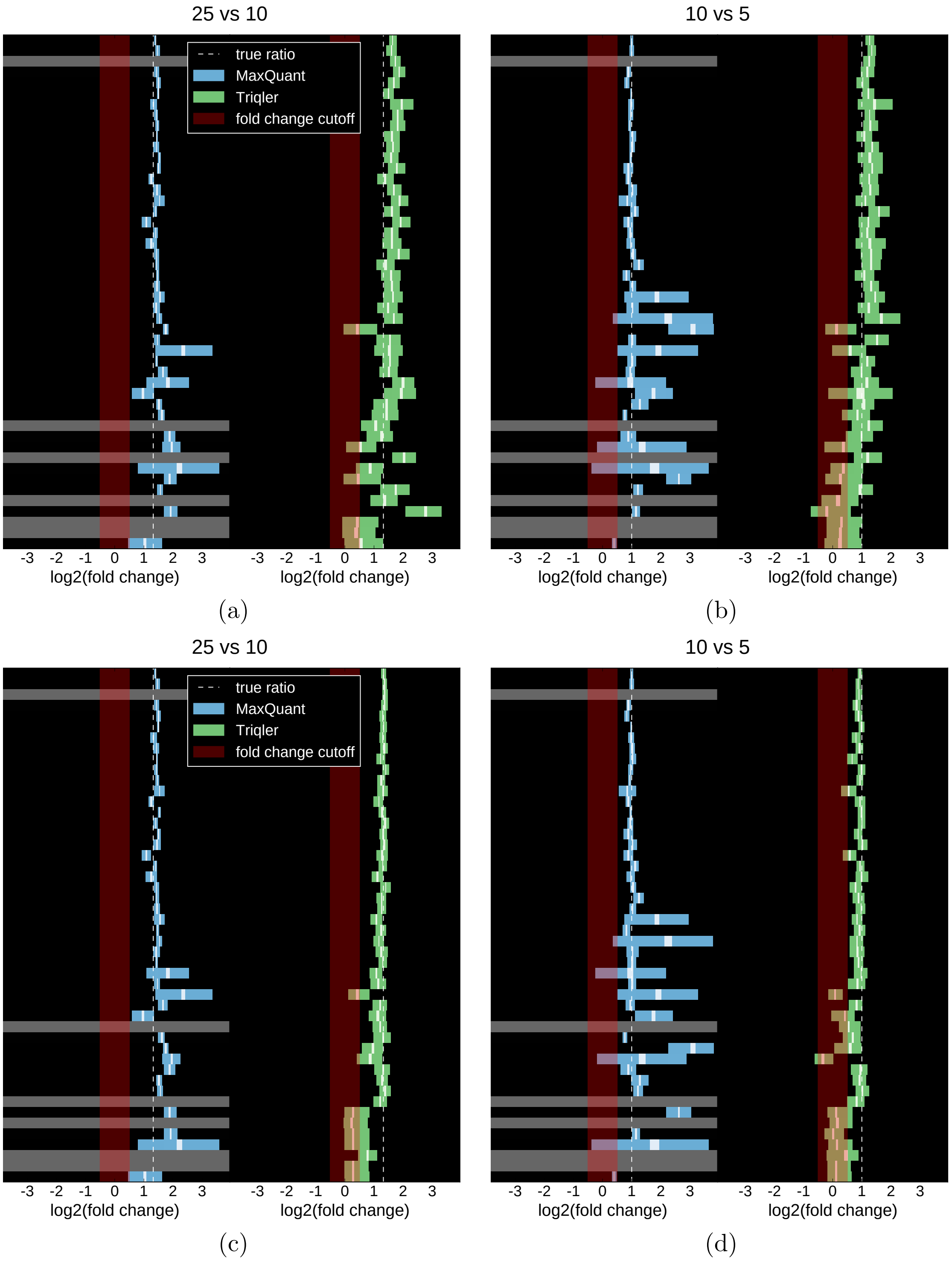

Supplementary Figure 8: **The posterior distributions for the fold change of the spiked-in UPS proteins follow the true spike-in ratios well.** We compare the uncertainty of the fold change estimations of MaxQuant+Perseus (blue) and Quandenser+Triqler (green). Each row represents a UPS protein sorted from top to bottom by protein FDR, where the colored portions represent the 90% and the white portions the 10% confidence/credible intervals for the 25 vs 10 fmol **(a)** and 10 vs 5 fmol comparisons **(b)**. The quantitative accuracy of Quandenser+Triqler can be further improved by using the peak apex intensity, instead of the peak summed intensity, at the cost of reduced sensitivity **(c)**, **(d)**.

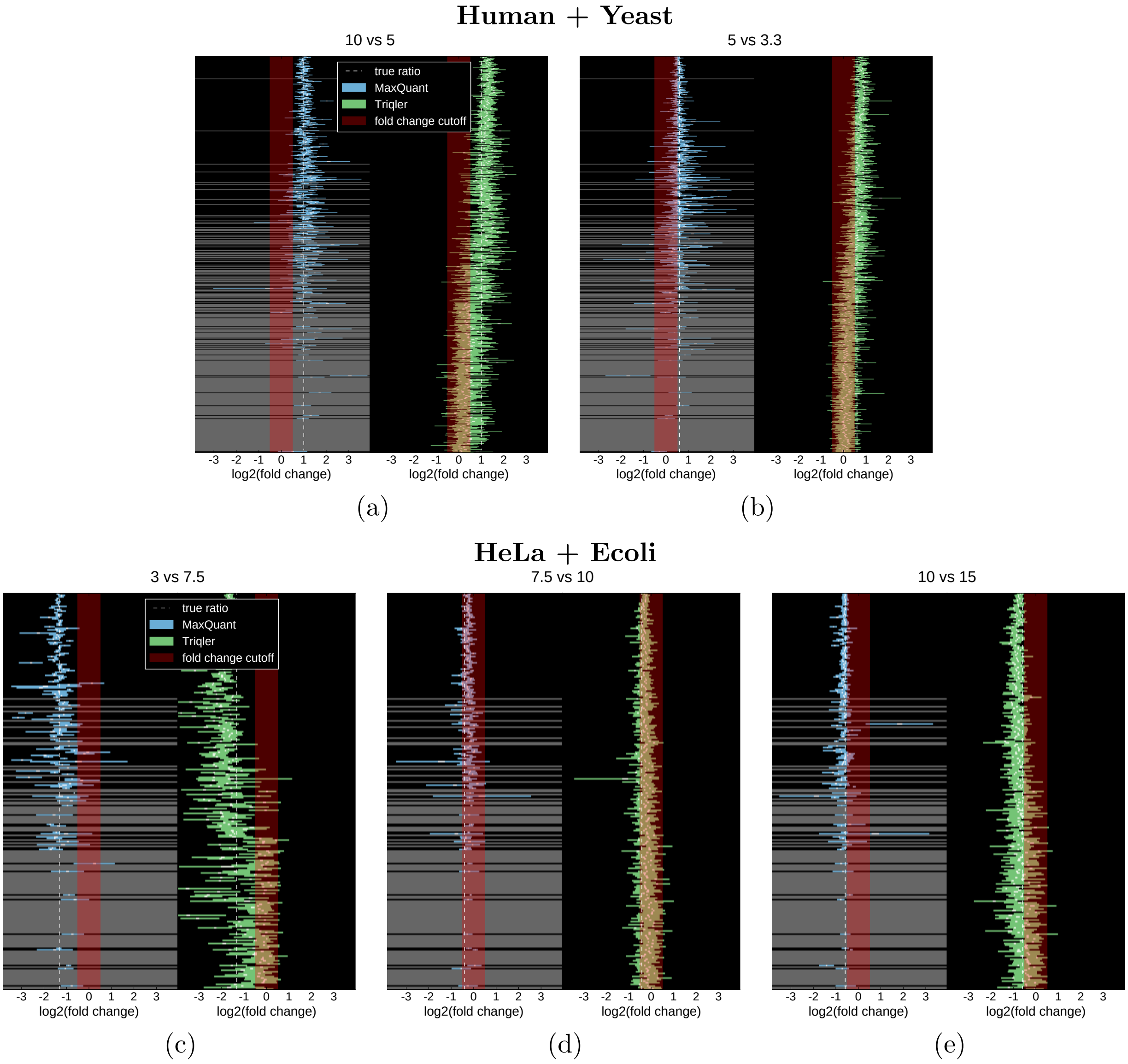

Supplementary Figure 9: **Quandenser+Triqler provides stable estimates of the fold changes for both the human-yeast (a, b)and HeLa+Ecoli proteome mixture sets (c, d, e).** We compare the uncertainty of the fold change estimations of MaxQuant+Perseus (blue) and Quandenser+Triqler (green). Each row represents a, respectively, yeast or e. coli protein sorted from top to bottom by protein FDR, where the colored portions represent the 90% and the white portions the 10% confidence/credible intervals.

Supplementary Note 4

Decoy features. We define an incorrect feature-feature match as a match where the two connected MS1 features do not originate from the same analyte. In a similar fashion to database searching, we model the incorrect matches by so-called decoy matches. Instead of reversing an amino acid sequence, we now ensure that the matched feature for a particular query feature is incorrect by searching in an m/z region offset by a fixed value of 5*1.000508 Th. The idea behind this offset is that the density, w.r.t. precursor m/z and retention time, of MS1 features is approximately the same in this offset region. This thus simulates the situation where a particular query feature is matched to a feature in the non-offset region if the truly matching feature is either not present, or if another incorrect feature is closer, by some weighting of the numeric features, such as precursor m/z and retention time difference.

To check the assumption that matches to decoy features mimic matches to incorrect features, we can plot the distribution of the numeric features and check if the distributions between target and decoy matches overlap in the region where we expect very few correct matches (Supplementary Figure 10). In our case, we see a good overlap between targets and decoys for large retention time and precursor m/z differences. For the precursor m/z differences, we observe a strange behavior in the $[(-)10, (-)50]$ ppm range, where there is a depletion of target features, which leads to conservative estimates of the FDR. We hypothesize this to be be due to peaks being aggregated within this window by the feature finding algorithm, Dinosaur.

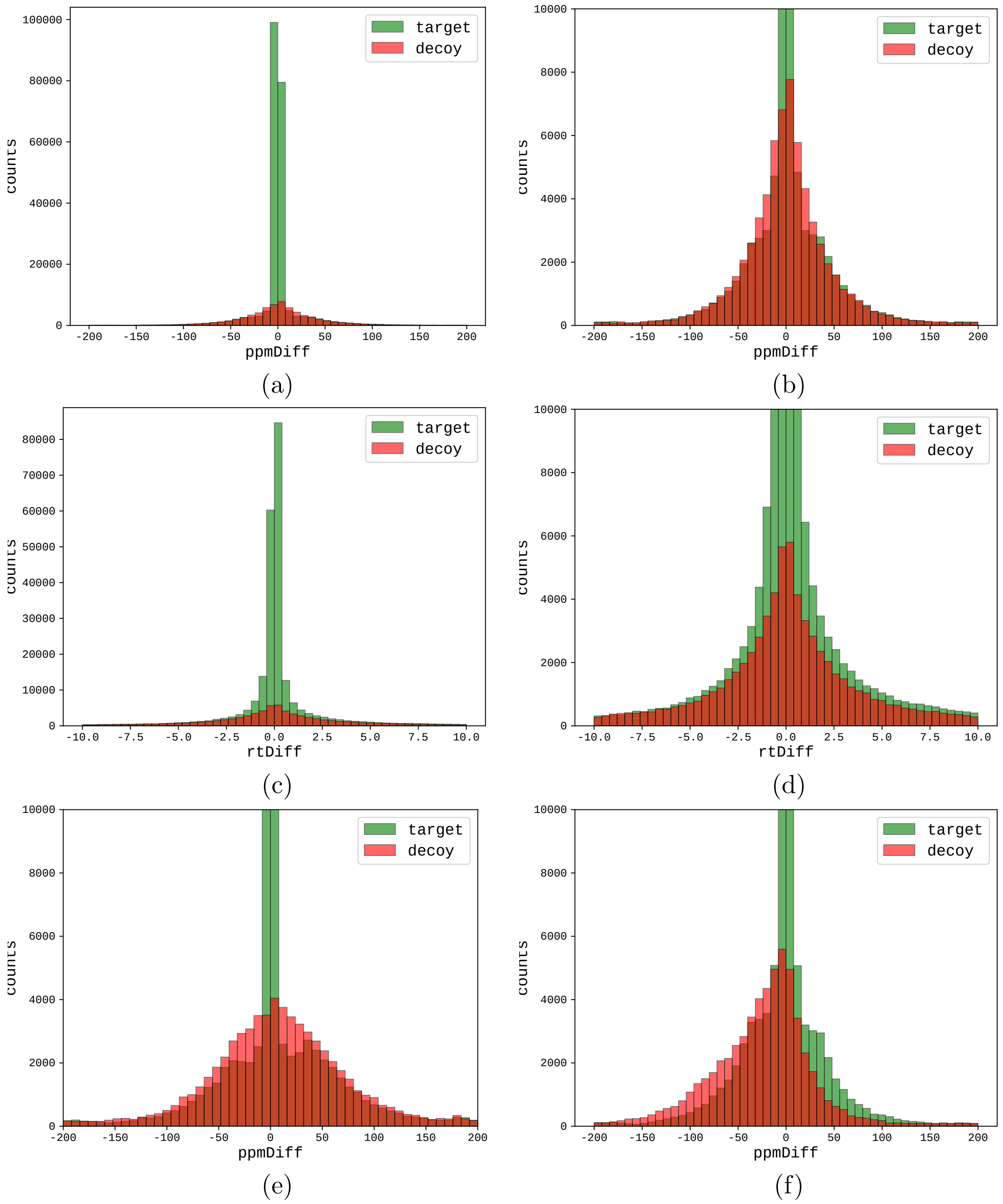

Supplementary Figure 10: **Decoy MS1 features provide a good proxy for incorrect target MS1 features.** **(a)** Histogram of the precursor m/z difference in parts-per-million (ppm) for all matches in a single pair-wise alignment in the Hela-Ecoli mixture set. We see a clear enrichment of target MS1 features close to zero and good overlap between target and decoy feature matches far from zero. **(b)** same as (a), but zoomed in to 10000 counts. **(c)** same as (a), but for aligned retention time differences in minutes. Again, we see enrichment close to zero. **(d)** same as (c) but zoomed in to 10000 counts. Again, overlap of target and decoy feature matches for large retention time differences can be observed. **(e)** same as (b), but for Dinosaur in targeted mode. Again, we see good overlap from around 50 ppm. **(f)** same as (b), but using a decoy precursor m/z offset of 5.025 instead of $5 \cdot 1.000508$, mimicking the offset used in DeMix-Q. The non-integer shift of the precursor mass causes a bias in the mass difference distribution because peptide masses have to be additions of amino acids masses.

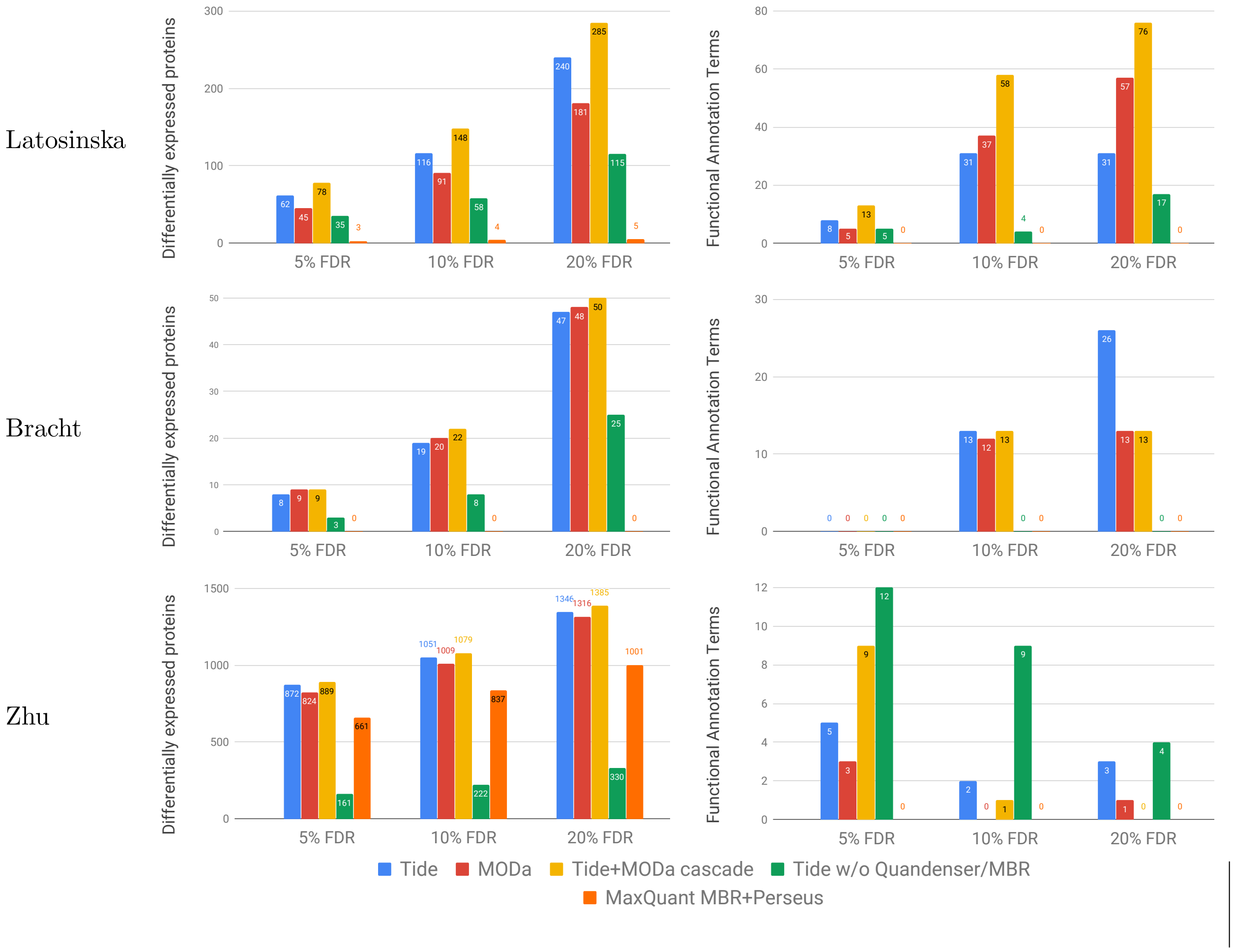

Supplementary Figure 11: **Benchmark of differentially abundant proteins and enriched functional annotation terms.** The analyzed methods were three quantification-first approaches, Quandenser+Triqler when using the search engine Tide (**blue**), MODa (**red**), and a cascade search with first Tide and subsequently MODa (**yellow**); and three identification-first approaches, Tide and Triqler without clustering and the match-between-runs (MBR) feature (**green**), and MaxQuant with MBR followed by statistical analysis with Perseus (**orange**) and EBRCT (**light blue**). Overall, we discovered more differentially abundant proteins and enriched functional annotation terms with than without Quandenser. Notably, we found enriched functional annotation terms for the Bracht set for which no enrichments were previously found. The left plots show the number of differentially abundant proteins at 3 differential abundance FDR thresholds. The plots on the right show the number of significant functional annotation terms we discovered with DAVID using the sets obtained in the left plots. Note that the FDR reported in the plots on the right refer to the differential abundance FDR and not the functional annotation term FDR, which was kept fixed at 5%.

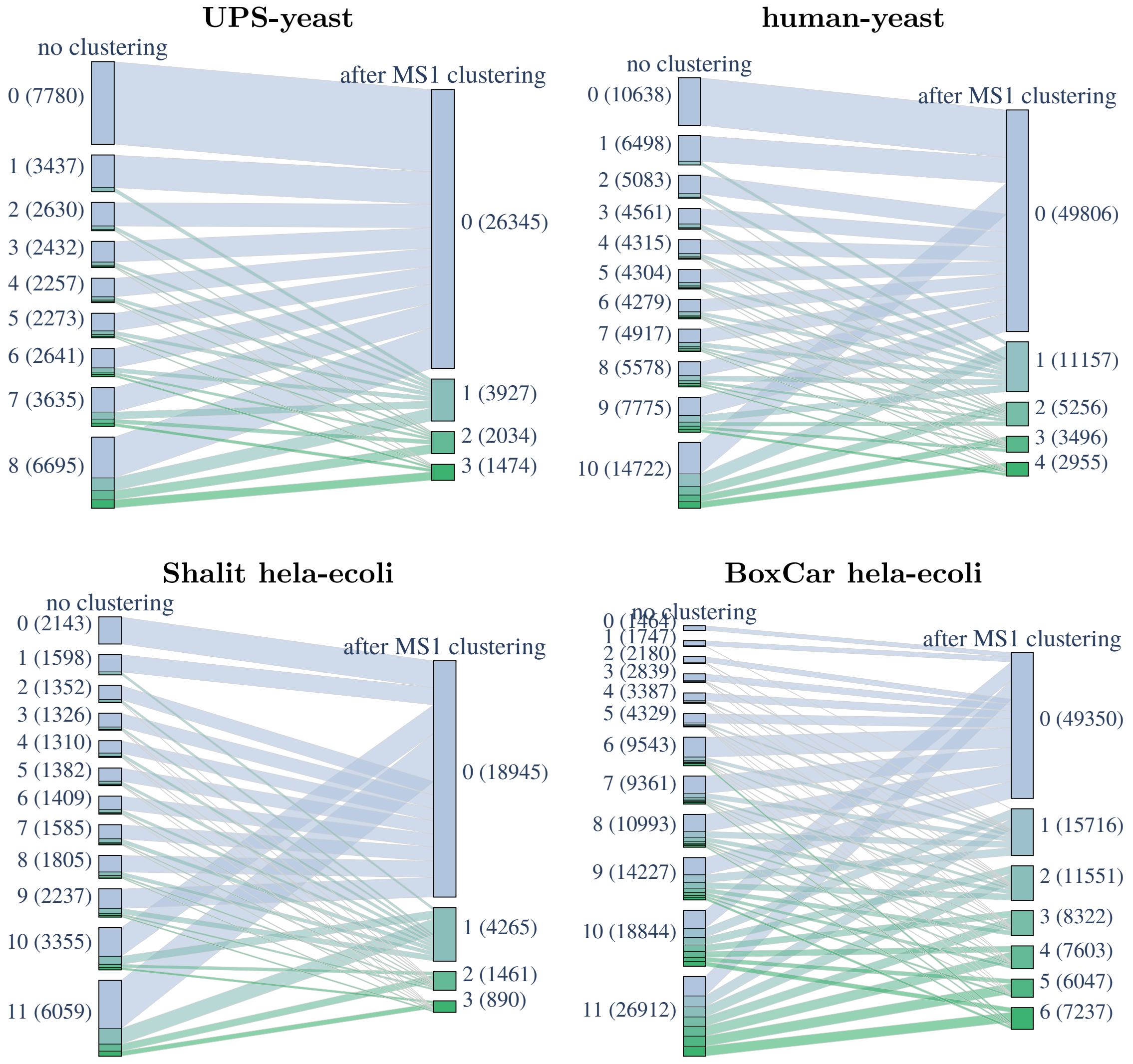

Supplementary Figure 12: **MS1 clustering drastically reduces the number of missing values.** For each of the engineered datasets, we plot the number of feature groups with MS2 spectrum with M missing values without clustering (left bars) and after MS1 clustering (right bars), annotated as "M (number of feature groups)". We see that even feature groups with an MS2 spectrum in only a single run (the bottom bar on the left in each panel) can often be rescued by MS1 clustering, frequently even leading to no missing values at all.

human-yeast

Method	10 vs 5		10 vs 3.3		5 vs 3.3	
	tp	fp	tp	fp	tp	fp
Quandenser+Tide+Triqler						
- max-missing 8	434	43	511	54	296	2
- max-missing 7	418	34	490	46	276	2
- max-missing 6	401	32	465	40	251	1
- max-missing 5	381	27	441	32	227	0
**- max-missing 4**	366	24	419	24	198	0
- max-missing 3	343	19	395	14	177	0

Supplementary Table 2: **Quandenser+Triqler achieves reasonable FDR control for the human-yeast set (11 runs in total), even with many allowed missing values.** The table lists the number of true and false positive significantly differentially abundant proteins at a 5% reported FDR threshold for different numbers of missing values for the human-yeast set using the Quandenser+Tide+Triqler pipeline. In the manuscript, max-missing 4 was used. As expected, sensitivity increases with more allowed missing values. The empirical FDR increases with higher number of allowed missing values as well and although it exceeds the reported 5% with more than 5 missing values, it stays below 10% even when allowing up to 8 missing values.

Supplementary Note 5

Analysis of increase in performance due to clustering. The inaccurately quantified peptides in Supplementary Figure 13 could give rise to falsely quantified proteins, especially if their quantifications would be considered as reliable as peptides before MS1 matching. Of the feature groups that were added by MS1 matching $4124/18458 = 22\%$ had a $|logFC| > 1.0$, for the feature groups before MS1 clustering, this rate was $2845/25681 = 11\%$. While the inaccurately quantified clusters might seem plentiful, by using our probabilistic graphical model, their influence is frequently down-weighted by considering matching probabilities and the agreement with other peptides of the protein. We indeed see a rise on protein level for the number of false positives (which are all human) from 3 to 14 after MS1 clustering for the 5vs10 comparison (Supplementary Table 3), but the observed differential expression FDR stays below the reported 5%, rising from 1.6% to 3.9%.

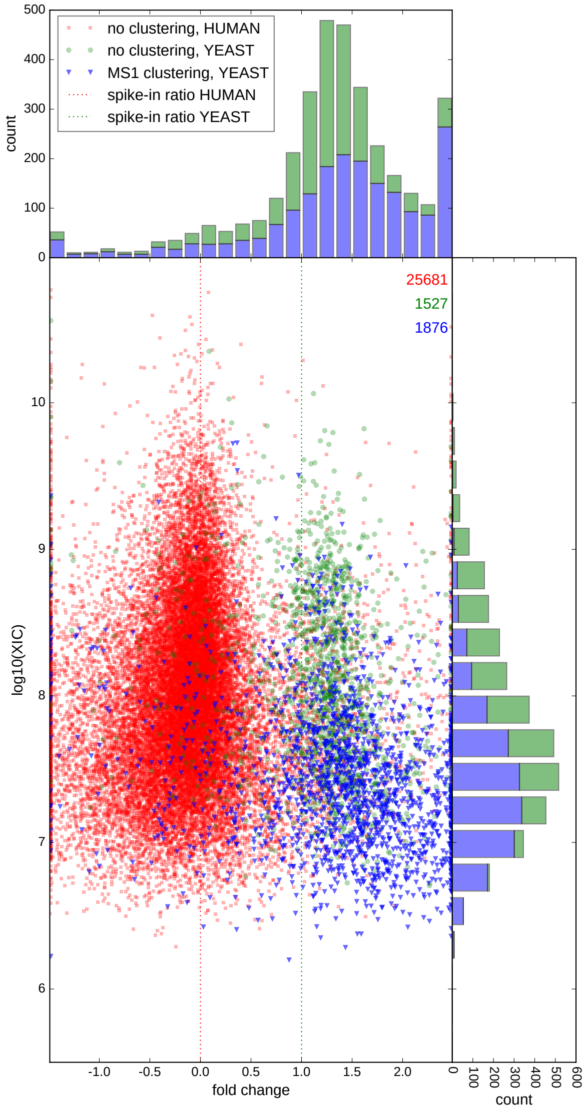

(a)

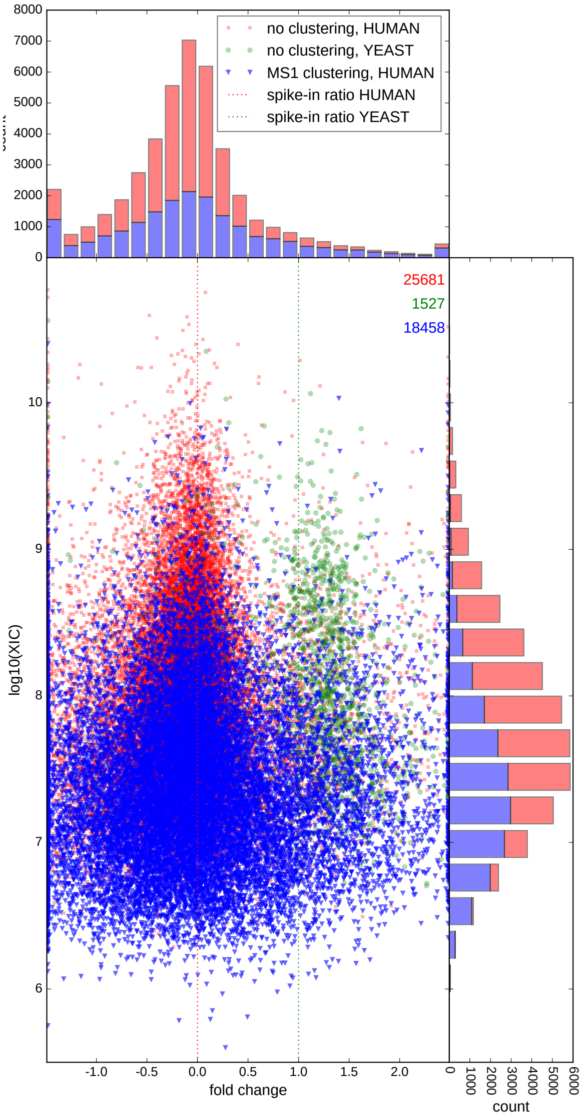

(b)

Supplementary Figure 13: **MS1 clustering (blue) almost doubles the number of peptides quantified at 1% FDR.** Scatter plots with histogram projections of peptide fold changes against extracted ion current (XIC) at 1% peptide-level identification FDR with at most 4 missing values for the human-yeast set. Fold changes are taken from predictions of the 10 vs 5 comparison, fold changes outside of the plotted range are set to the respective minimum or maximum fold change within the plotted range. We see a separation of human (red) and yeast peptides (green) before applying any clustering, which follow the spike-in ratios. We demonstrate that MS1 clustering more than doubles the number of yeast peptides **(a)** and adds around 70% more peptides for human **(b)**. These added peptides follow the expected fold changes and show a clear enrichment for lower XIC values, contributing to lowering the limit of detection (LOD).

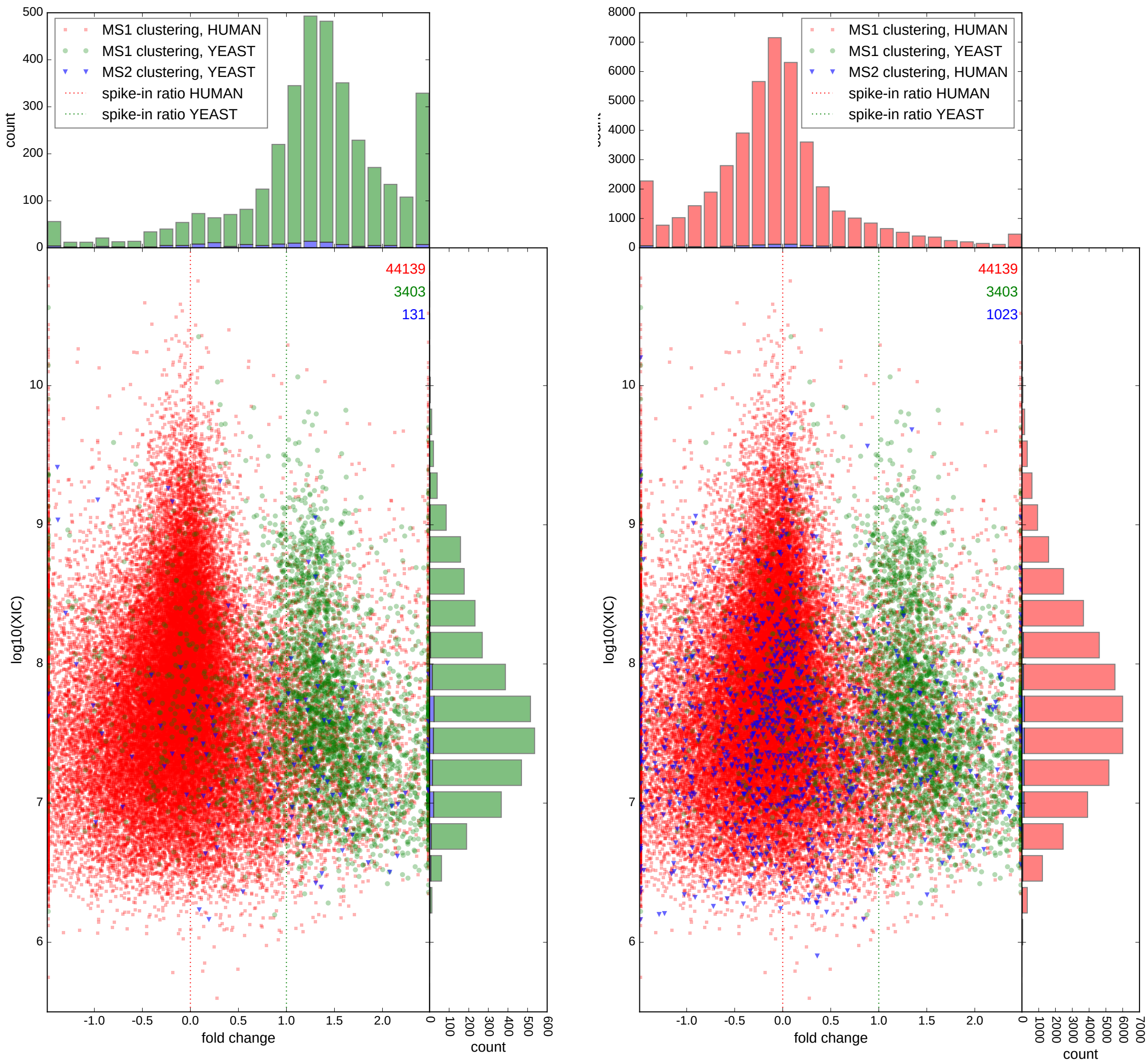

(a) (b)

Supplementary Figure 14: **MS2 clustering (blue) only modestly increases the number of peptides quantified at 1% FDR.** Scatter plots with histogram projections of peptide fold changes against extracted ion current (XIC) at 1% peptide-level identification FDR with at most 4 missing values for the human-yeast set. Fold changes are taken from predictions of the 10 vs 5 comparison, fold changes outside of the plotted range are set to the respective minimum or maximum fold change within the plotted range. We see that human (red) and yeast peptides (green), with MS1 clustering but without MS2 clustering, follow the spike-in ratios. MS2 clustering only adds 4% more yeast peptides **(a)** and 2% more peptides for human **(b)**. Since MS1 clustering already allows identity propagation between features, the contribution of MS2 clustering is limited to reducing the number of hypotheses tested and increasing signal on noisy MS2 spectra through the formation of consensus spectra.

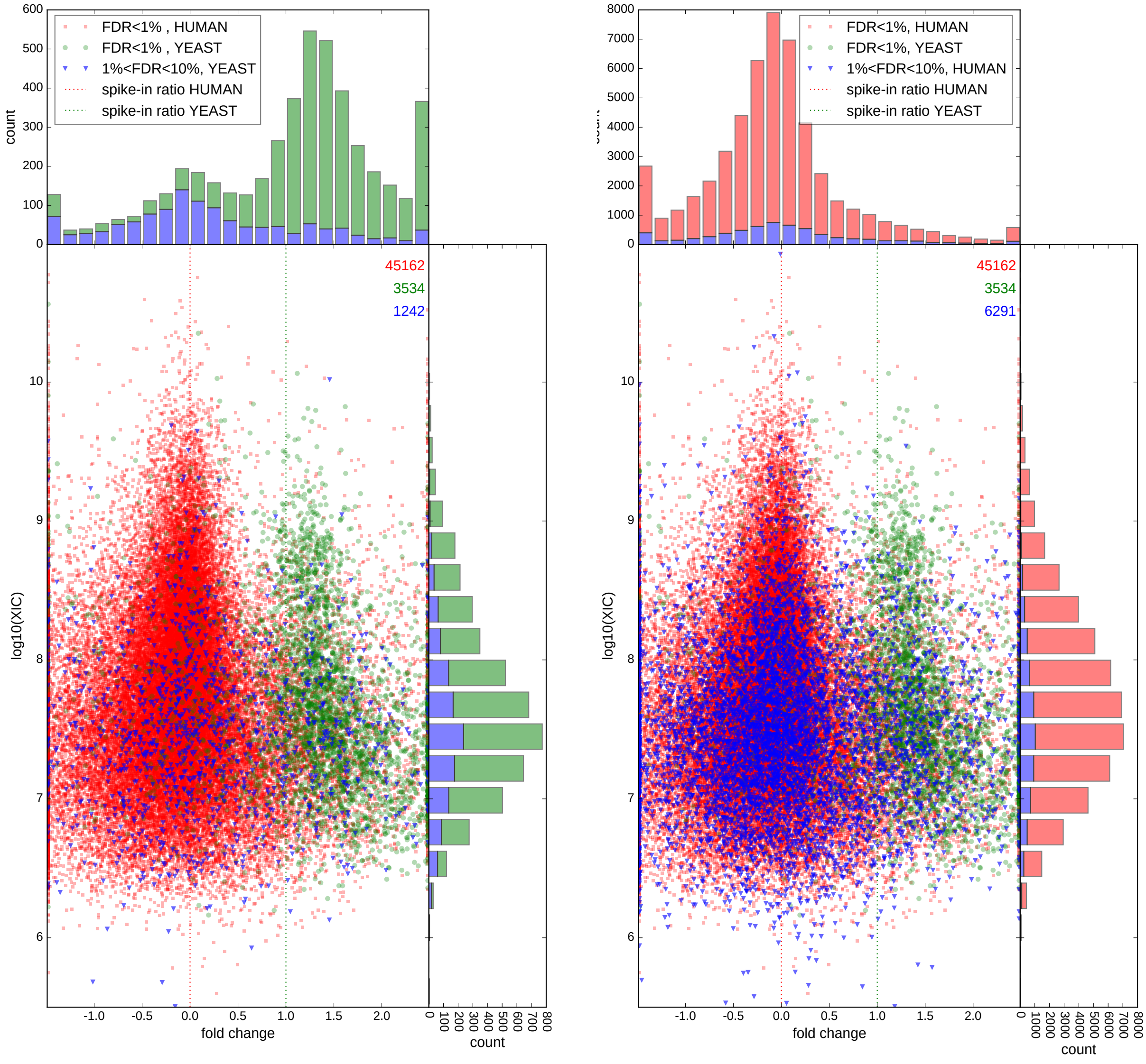

(a) (b)

Supplementary Figure 15: **Peptide identification between 1% and 10% FDR (blue) could provide valuable extra information that is typically discarded.** Scatter plots with histogram projections of peptide fold changes against extracted ion current (XIC) at 1% peptide-level identification FDR with at most 4 missing values for the human-yeast set. Fold changes are taken from predictions of the 10 vs 5 comparison, fold changes outside of the plotted range are set to the respective minimum or maximum fold change within the plotted range. We see a separation of human (red) and yeast peptides (green), with MS1 and MS2 clustering, which follow the spike-in ratios. We see that although many peptides identified as coming from yeast between 1% and 10% FDR are most likely misidentified as they follow the human spike-in ratio, a non-negligible amount of peptides do follow the correct spike-in ratio of yeast as well **(a)**. For the majority class of human, this looks better on first sight, but will probably still contain many false positives **(b)**. Even though the peptide identification between 1% and 10% FDR should be handled with the greatest caution, they could help to confirm or oppose evidence below the 1% FDR threshold.

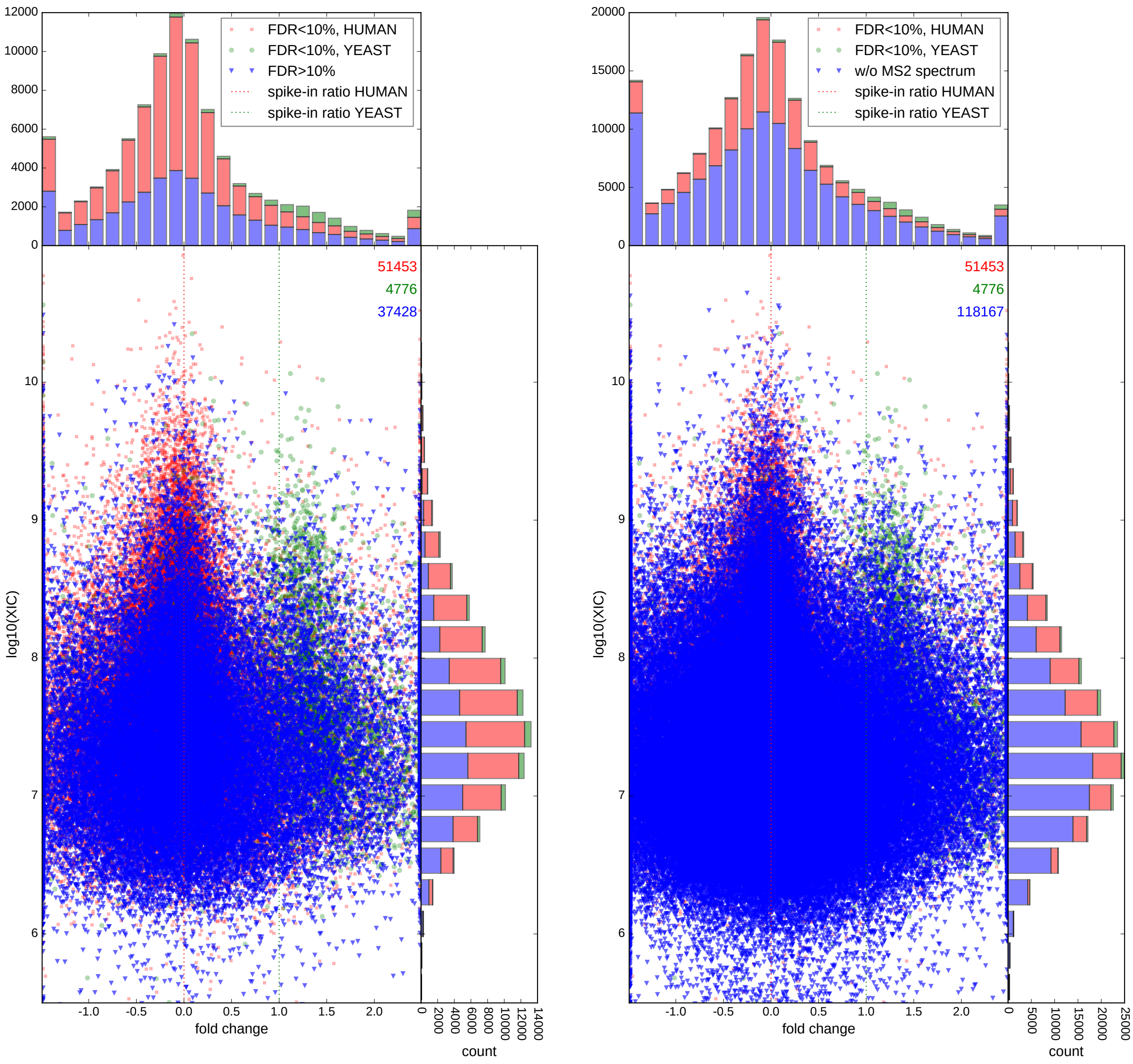

(a) (b)

Supplementary Figure 16: **MS1 feature groups without identification (left, blue) and without MS2 spectrum (right, blue) represent a sizeable part of the proteome that remains unknown.** Scatter plots with histogram projections of peptide fold changes against extracted ion current (XIC) at 10% peptide-level identification FDR with at most 4 missing values for the human-yeast set. Fold changes are taken from predictions of the 10 vs 5 comparison, fold changes outside of the plotted range are set to the respective minimum or maximum fold change within the plotted range. We see a separation of human (red) and yeast peptides (green), with MS1 and MS2 clustering, which follow the spike-in ratios. MS1 feature groups without identification at 10% FDR still make up around 40% of the data **(a)**. MS1 feature groups without MS2 spectrum almost triples the data, which in the future might be identifiable by, e.g. accurate mass and retention time predictions **(b)**.

UPS-yeast

Method	Num feature groups UPS	yeast	25 vs 10 tp	fp	25 vs 5 tp	fp	10 vs 5 tp	fp
No clustering, Tide, FDR < 1%	230	9193	37	0	38	0	33	1
+ MS1 clustering	(+139) 369	(+2956) 12149	44	2	46	2	39	0
+ MS2 clustering	(+4) 373	(+322) 12471	43	2	45	2	38	0
+ 1% < FDR < 10%	(+50) 423	(+3115) 15595	43	2	46	2	38	2
+ FDR > 10%	(+34166) 50184		43	1	46	0	37	0
+ no MS2 spectrum	(+70542) 120726		-	-	-	-	-	-

human-yeast

Method	Num feature groups yeast	human	10 vs 5 tp	fp	10 vs 3.3 tp	fp	5 vs 3.3 tp	fp
No clustering, Tide, FDR < 1%	1527	25681	182	3	205	1	85	0
+ MS1 clustering	(+1876) 3403	(+18458) 44139	346	14	385	21	176	0
+ MS2 clustering	(+131) 3534	(+1023) 45162	346	16	388	22	178	0
+ 1% < FDR < 10%	(+1242) 4776	(+6291) 51453	366	24	419	24	198	0
+ FDR > 10%	(+37428) 93657		366	24	419	24	198	0
+ no MS2 spectrum	(+118167) 211824		-	-	-	-	-	-

Shalit hela-ecoli

Method	Num feature groups E. coli	HeLa	3 vs 7.5 tp	fp	3 vs 10 tp	fp	3 vs 15 tp	fp	7.5 vs 10 tp	fp	7.5 vs 15 tp	fp	10 vs 15 tp	fp
No clustering, Tide, FDR < 1%	269	5836	44	0	47	0	52	0	0	0	48	0	41	0
+ MS1 clustering	(+796) 1065	(+7625) 13461	138	0	146	1	176	0	0	0	161	0	112	0
+ MS2 clustering	(+35) 1100	(+613) 14074	142	0	150	1	183	0	0	0	167	0	116	0
+ 1% < FDR < 10%	(+399) 1499	(+4551) 18625	159	2	167	2	206	0	0	0	185	1	137	0
+ FDR > 10%	(+38232) 58356		190	7	198	4	229	0	0	0	194	3	138	0
+ no MS2 spectrum	(+156684) 215040		-	-	-	-	-	-	-	-	-	-	-	-

BoxCar hela-ecoli*

	Num feature groups		BoxCar 2 vs 12				Shotgun 2 vs 12			
			1-sided test		2-sided test		1-sided test		2-sided test	
Method	E. coli	HeLa	tp	fp	tp	fp	tp	fp	tp	fp
No clustering, Tide, FDR < 1%	2626	17269	460	14	460	21	475	8	475	40
+ MS1 clustering	(+6665) 9291	(+34324) 51593	999	14	999	54	977	3	977	176
+ MS2 clustering	(+262) 9553	(+1543) 53136	1007	14	1007	53	982	3	982	186
+ 1% < FDR < 10%	(+1486) 11039	(+10735) 63871	1027	18	1027	68	999	3	999	216
+ FDR > 10%	(+62175) 137085		1029	18	1029	71	1002	4	1002	234
+ no MS2 spectrum	(+229680) 366765		-	-	-	-	-	-	-	-

* As the BoxCar runs rely on identification propagation from the Shotgun runs, no representative results could be generated from the separate analyses, as was done in the rest of the manuscript. Therefore, results are shown for a combined analysis of the 6 BoxCar with the 6 Shotgun runs with maximum allowed missing values, $M = 6$.

Supplementary Table 3: **MS1 clustering is the biggest contributor to the increased sensitivity of our pipeline.** By filtering the feature groups that are added by certain steps of our pipeline, we could look at the contributions of these steps. MS1 clustering clearly shows the biggest contribution to sensitivity, whereas contributions of MS2 clustering and including less confident peptides is rather modest.

Supplementary Note 6

The analyzed datasets are characterized by the following parameters.

M: max missing values
fc_{eval}: $|\log_2|$ fold change eval for Triqler
MS2 sp: number of MS2 spectra
SFP: number of spectrum-feature pairs, number of spectra that need to be searched by the search engine
FG: feature groups
FG sp: number of feature groups with at least one MS2 consensus spectrum
cs sp.: number of consensus spectra
cs SFP: consensus spectrum-feature pairs, number of spectra that need to be searched
rd SFP: reduction in number of spectrum-feature pairs that need to be searched

Dataset	PRIDE ID	runs	M	fc_{eval}	MS2 sp	SFP	FG	FG sp	cs sp	cs SFP	rd SFP
UPS-yeast	PXD002370	9	3	0.8	535k	934k	121k	51k	61k	109k	88%
human-yeast	PXD007683	11	4	0.5	807k	1.12M	212k	94k	116k	178k	84%
Shalit hela-ecoli	PXD001385	12	3	0.5	340k	533k	215k	53k	67k	96k	82%
BoxCar hela-ecoli	PXD006109	12	6	0.5	629k	1.02M	370k	124k	134k	218k	79%
Latosinska	PXD002170	8	4	0.8	413k	991k	83k	47k	122k	183k	82%
Bracht	PXD001474	27	7	0.5	1.01M	1.47M	69k	45k	106k	150k	90%
Zhu	PXD006847	18	11	1.0	593k	1.02M	73k	52k	117k	187k	82%

Supplementary Table 4: **Summary of the datasets, including used parameters and reduction in searched MS2 spectra.**
