# Supplementary material for: Focus on the spectra that matter by clustering of quantification data in shotgun proteomics
Source: Nat Commun. 2020 Jun 26;11:3234. doi: 10.1038/s41467-020-17037-3 (PMC7319958; doi:10.1038/s41467-020-17037-3)
Supplement: Supplementary file 2 — Description of Additional Supplementary Files [file 41467_2020_17037_MOESM2_ESM.docx]

**Description of Additional Supplementary Files**

**File Name: Supplementary Data 1**

**Description:** Tentative identifications for spectrum clusters of potential interest in the UPS-yeast dataset using de novo and open modification searches. The first table focuses on the largest unidentified spectrum clusters, whereas the second table uses the cosine similarity to the UPS expression pattern as a criterion for interest. The third table lists the fragment ions of 5 consensus spectra from the second table that seem to stem from carbohydrates instead of amino acid oligomers.

**File Name: Supplementary Data 2**

**Description:** Significant enriched functional annotation terms. The enriched functional annotation terms among the significant proteins at 3 differential expression FDR thresholds (5, 10 and 20%), as reported by Triqler, for the respective search results from Tide, MODa and a cascaded search consisting of Tide followed by MODa on the Latosinska, Bracht and Zhu dataset. The p-values reported for the functional annotation terms are calculated using the Fisher's exact test. For reporting the number of significant terms at 5% FDR, we used the reported values after multiple testing correction by the Benjamini-Hochberg procedure, as reported in the "Benjamini" column.
